# The Subjective Experience of Autobiographical Remembering: Conceptual and Methodological Advances and Challenges

**DOI:** 10.3390/jintelligence12020021

**Published:** 2024-02-12

**Authors:** Carlo Chiorri, Manila Vannucci

**Affiliations:** 1 Department of Educational Sciences, University of Genoa, Corso A. Podestà 2, 16128 Genova, Italy; 2 Department of Neurofarba, Section of Psychology, University of Florence, Via San Salvi 12, Padiglione 26, 50135 Florence, Italy

**Keywords:** phenomenology, autobiographical memory, retrieval process

## Abstract

The investigation of the phenomenology of autobiographical memories (i.e., how a memory is subjectively experienced and its meaning) has provided an important contribution to our understanding of autobiographical remembering. Over the last two decades, the study of phenomenology has received widespread scientific attention, and the field has undergone quite relevant conceptual and methodological changes. In the present work, we (1) review some basic and well-established research findings and methodological achievements; (2) discuss new theoretical and methodological challenges, with a special focus on the issue of the phenomenological experience of the retrieval process and its relationship with the phenomenology of the products of retrieval; and (3) propose an alternative way of conceptualizing and understanding it in the framework of experimental phenomenology.

## 1. Introduction

*“As I remember it, I can see it very clearly in my mind. I can also feel the emotions I felt when it occurred”; “It is as if I am reliving that event”; “I believe the event in my memory really occurred as I remember it”*

Sentences like these are often used when we talk about our autobiographical memory (ABM), that is, when remembering our personal past. Specifically, experiences like seeing with the mind’s eye, reliving an event, or believing in the accuracy of one’s own memory refer to different dimensions of the phenomenology of autobiographical remembering.

Although the word “phenomenology” has been used in slightly different ways (for a discussion, see Vanaken et al. 2022), in the field of ABM, phenomenology is mostly used to indicate the multifaced *subjective experience* associated with remembering one’s own personal past; that is, how memories appear in one’s own conscious experience, the ways they are experienced, and their personal meaning. In the investigation of the phenomenology of ABM, the focus is not on “what” (that is, the content of the memory) or the number of memories recalled, but on “how” a memory is subjectively experienced and its meaning. Although phenomenology also accompanies the retrieval of conceptual and semantic ABMs (i.e., autobiographical knowledge) (e.g., Klein and Markowitsch 2015; Renoult et al. 2012), a specific phenomenology characterizes episodic (i.e., event-specific knowledge) ABMs. The configuration of phenomenal properties that gives rise to the feeling of re-living or re-experiencing the past (a mental state that Tulving (1985) has termed “autonoetic awareness”) is the hallmark of ABM (e.g., Conway 2005; Tulving 1985, 2002). In the present paper, we aim to focus on and discuss (i) some basic and well-established research findings and methodological achievements and (ii) the recent developments and challenges facing this research field.

As we will show in the following, in examining the contribution of phenomenology to our understanding of ABM, we should keep in mind that, for a long time, mainstream cognitive research on ABM has been focused almost exclusively on the investigation of *voluntary (intentionally) retrieved* ABMs (i.e., memories of personal events intentionally generated in response to cues or instructions provided by the experimenter), and intentional retrieval has been supposed to occur mainly through a generative, effortful, and time-consuming process. During the past decade, increasing evidence has been reported showing that, in the context of voluntary retrieval, ABMs are frequently recalled in a direct, effortless way (e.g., Uzer 2016; Uzer and Brown 2017; Uzer et al. 2012). Furthermore, the experimental investigation of ABM retrieval has been extended to *involuntary (spontaneous) autobiographical memories* (IAMs; i.e., memories of personal events that come to mind with no conscious or deliberate attempt directed at their retrieval; see, e.g., Berntsen 2010, 2021).

These important developments have stimulated research on the phenomenological properties of memories recalled under different retrieval conditions and a more general interest in the investigation of the subjective experience of the *retrieval process itself*, that is, what “how” retrieving a memory is like and the “feelings” experienced during the retrieval—instead of the phenomenology of the final (memory) products. In this regard, very recently, some researchers (Moulin et al. 2023; see also the contribution of philosophers of memory, Perrin et al. 2020; Perrin and Sant’Anna 2022) have explicitly called for a more systematic empirical investigation of the phenomenology of the retrieval process, as has been performed with the phenomenological characteristics of ABMs. As we shall discuss, these recent advances open up new avenues of research that come with relevant theoretical and methodological implications that need to be systematically addressed in the future. Relevant to the aims of this special issue, we suggest an analogy of the phenomenology of the retrieval process with that of perception traditionally studied by experimental phenomenology that might inspire an alternative way of conceptualizing and understanding it.

## 2. Phenomenology of Autobiographical Memories: Types of Memories and Individual Differences

The investigation of the phenomenology of ABM has provided an essential contribution to our understanding of the complex and flexible nature of autobiographical remembering, and the dimensions of the phenomenological experiences associated with remembering have received much attention in the theoretical models of ABM (Conway 2005; Rubin 2005, 2006).

Over the past three decades, the issue of the phenomenology of ABMs has received widespread scientific attention, with an increase in interest in philosophy, psychology, and neuroscience (see, for a review on the neural bases of the phenomenology of ABMs, Simons et al. 2022), and different research trends have emerged (e.g., investigation of the phenomenology from a life-span perspective, investigation of phenomenological changes associated with clinical and neurological disorders). In spite of this diversification of the research field, we can identify some basic and well-established research findings. Studies have consistently shown that (i) memory and event features and (ii) individual differences in psychological functioning among those who remember affect the phenomenology of ABM.

Regarding *memory and event features*, clear phenomenological differences have been found between (a) true and false memories (e.g., Johnson et al. 1988; Heaps and Nash 2001); (b) self-defining memories and earliest memories (Montebarocci et al. 2014); (c) memories for recent and remote events (e.g., Eich et al. 2012; Nigro and Neisser 1983; Sutin and Robins 2007); (d) memories for unique events and memories of repeated events (e.g., Peterson et al. 2016; Waters et al. 2014); and (e) memories for positive, negative, and neutral events (e.g., D’Argembeau et al. 2003; Maki et al. 2013).

For example, in one of the seminal studies, Johnson et al. (1988) showed that true memories (memories for perceived events) differed from false memories (memories for imagined events) in a relevant number of phenomenological dimensions (assessed by the Memory Characteristics Questionnaire (MCQ), see Table A1 in Appendix A). Specifically, memories of real events had higher ratings for all phenomenological dimensions related to perceptual characteristics (details, vividness) and contextual information (location, time) compared to memories of imagined events. In addition, participants used the differences in these qualitative characteristics to support their belief in the origin of memory, suggesting that specific phenomenological characteristics enable participants to distinguish a true memory from a false one.

The phenomenological profile of different types of ABMs may also reflect their specific roles for the current self and identity. Studies that directly compared the phenomenological characteristics of self-defining memories and earliest childhood memories have made an important contribution in this regard (Montebarocci et al. 2014; Sutin and Robins 2007). Self-defining memories are memories of events that are extremely important to identity processes (Blagov and Singer 2004; Singer et al. 2007); they support self-consistency and self-coherence, particularly in tumultuous or challenging transitional periods (Conway et al. 2004). In line with this function, Montebarocci et al. (2014) found that self-defining memories were rated as more vivid, coherent, rich in sensory details, and clear in time perspective compared to earliest childhood memories. Moreover, they were more emotionally intense, more likely to be seen from a first-person visual perspective, and more likely to be shared with other people.

Consistent differences in several aspects of phenomenology also emerged when memories of distant (childhood) or remote events were compared to memories of recent ones (see, e.g., Luchetti and Sutin 2018; Sutin and Robins 2007). For example, Sutin and Robins (2007) found significant differences between the two types of memories on all the phenomenological dimensions assessed in the study (by the Memory Experiences Questionnaire, see Table A1, Appendix A): Recent memories were associated with a more intense phenomenological experience, being rated as more vivid and detailed, coherent, with a clearer time perspective, more accessible, more emotionally intense and positive, and more likely to be shared and recalled from the first-person visual perspective.

The investigation of the phenomenology of ABMs has also revealed *individual differences* in the subjective experience associated with autobiographical remembering, such as differences in the extent to which people can relive personal experiences, as well as differences in the reported characteristics of memories (e.g., their vividness and richness of details). A series of studies with healthy young adults have shown that certain aspects of phenomenology are affected by individual differences in psychological dimensions, such as personality (e.g., Rasmussen and Berntsen 2010; Rubin and Siegler 2004; Sutin and Robins 2005, 2010), emotion regulation (e.g., Fernández-Pérez et al. 2023; Pascuzzi and Smorti 2017), attachment styles (e.g., Sutin and Gillath 2009), self-esteem (e.g., Christensen et al. 2003; Sutin and Robins 2007), and visual imagery (e.g., Aydin 2018; D’Argembeau and Van der Linden 2006; Greenberg and Knowlton 2014; Vannucci et al. 2016; Vannucci et al. 2020).

In a pioneering study, Rubin and Siegler (2004) assessed individual differences in personality and, using the Autobiographical Memory Questionnaire (AMQ; see Table A1, Appendix A), evaluated several dimensions of the phenomenology of ABMs generated in response to cue words. The authors found that certain aspects of personality correlated with certain properties of phenomenology: Openness to feelings was strongly and positively associated with measures of belief in the accuracy of memories, the sense of recollection, the amount of sensory details, and the feeling of emotions while remembering. On the other hand, agreeableness, conscientiousness, and neuroticism were negligibly associated with the properties of phenomenology.

The results of subsequent studies confirmed the role of individual differences in personality in shaping the phenomenology of autobiographical remembering but also suggested that the association might be, at least in part, moderated by the types of memories. For example, Blagov et al. (2022) found a different pattern of results by asking participants to generate self-defining memories. Specifically, they reported that a feature of self-defining memories (explicit meaning-making) moderated the association between another feature (affect) and chronic emotional distress.

On the cognitive side, much attention has been paid to the association with individual differences in visual imagery. It is well known, from behavioral and neuroscientific studies on healthy participants, as well as from lesion studies, that visual imagery plays a crucial role in autobiographical retrieval (see, e.g., Conway 1990; Daselaar et al. 2008; Greenberg et al. 2005; El Haj et al. 2014, 2017). Moreover, visual imagery contributes to several phenomenological properties of ABM, such as vividness of memories (e.g., Brewer 1986; Greenberg and Rubin 2003), memory specificity (e.g., Williams et al. 1999), and the subjective experience of reliving during retrieval (e.g., El Haj et al. 2016; Greenberg and Rubin 2003).

Research on individual differences revealed a clear association between some aspects of phenomenology and individual differences in the ability/preference, and frequency of use of visual object imagery (i.e., the object imagery system processes the visual appearance of objects and scenes in terms of their shape, color information, and texture). Studies have found that higher levels of visual object imagery were predictive of a more intense autonoetic experience, that is, a greater amount of sensory details in memory, a stronger feeling of recollection, and a stronger experience of both sensory and emotional reliving (“seeing and feeling” as in the original event). In contrast, individual differences in spatial imagery were not significantly related to any of the phenomenological dimensions evaluated in the studies (Aydin 2018; Vannucci et al. 2020). This pattern of results has been replicated in two independent studies on different types of ABMs (e.g., asking participants to generate two personal past events from different time frames in Aydin 2018; asking participants to generate ABMs to cue words in Vannucci et al. 2020) and using different instruments to assess the phenomenology of ABM (e.g., selected items adapted from the MCQ and the Autobiographical Memory Questionnaire (AMQ) in Aydin 2018; the Phenomenology of Autobiographical Memory questionnaire (APAM) in Vannucci et al. 2020).

Changes in the phenomenology of ABM were also found to be linked to individual differences in subjective wellbeing and psychological distress (Luchetti et al. 2016; Sutin and Gillath 2009; Luchetti and Sutin 2016; Sutin et al. 2021) and mental health (see, e.g., Rottenberg et al. 2005). Distress levels have been linked to the retrieval of memories that are less phenomenologically powerful (less vivid, coherent, and detailed; Luchetti and Sutin 2016). In contrast, recalling phenomenologically rich personal events may enhance one’s sense of overall wellbeing and life satisfaction (e.g., Latorre et al. 2013; Sutin et al. 2021; Waters and Fivush 2015). In this regard, Sutin et al. (2021) found a pattern of association between the phenomenology of a recent memory (related to the pandemic) and a specific dimension of eudaimonic wellbeing, that is, the sense of purpose in life. Specifically, individuals with a higher sense of purpose in life retrieved ABMs that were generally more phenomenologically rich (e.g., more vivid, coherent, accessible, shared with others, with a clear time perspective and many sensory details) than participants lower in purpose in life. Interestingly, purpose in life was also associated with more positive affect and less negative affect during retrieval.

## 3. Assessment of the Phenomenology of Autobiographical Memories Using Comprehensive Measures

All researchers interested in the phenomenology of ABM agree that the subjective experience associated with autobiographical remembering is multifaceted. 

For a long time—and, although less frequently, still nowadays—studies in which the assessment of phenomenology was not the main aim evaluated the phenomenological experiences with a relatively small number of potentially relevant dimensions, with a preference for the reported quality of remembered material (e.g., vividness of memory, richness of sensory and contextual details, and memory specificity). Since the late 1980s, researchers have aimed at comprehensively mapping the range of phenomenological experiences (i.e., to fully describe the multifaced experiences of autobiographical remembering and its meaning to those who remember) and at developing psychometrically sound measures of such experiences.

Some standardized instruments for the assessment of ABMs have been developed to assess ABM deficits in clinical populations and include the Autobiographical Memory Interview (Kopelman et al. 1990), the Autobiographical Interview (Levine et al. 2002), and the Autobiographical Memory Test (Williams and Broadbent 1986; Williams et al. 2007). These tests require a long administration time because they frequently involve face-to-face interviews, followed by in-depth coding of the respondents’ verbal reports. As a consequence, to be reliable, the scores need good inter-rater agreement. Additionally, tests created to look for impairments in clinical populations have a strong tendency to exhibit ceiling effects in nonclinical participants (Kirk and Berntsen 2018).

On the other hand, the self-reported comprehensive measures of the phenomenological characteristics of ABM are basically of two types: They either present participants with some cue words (e.g., “city”, “dress”, “plant”) to elicit memory and ask them to evaluate the characteristics of the memory on a wide range of dimensions represented by a single item (Memory Characteristics Questionnaire (MCQ), Johnson et al. 1988; Autobiographical Memory Questionnaire (AMQ), Rubin et al. 2003; Autobiographical Memory Questionnaire (APAM), Vannucci et al. 2020), or they consider phenomenological properties as constructs and operationalize them by more items (Memory Experiences Questionnaire (MEQ), Sutin and Robins 2007; a short version of MEQ, Luchetti and Sutin 2016; Autobiographical Memory Characteristic Questionnaire (AMCQ), Boyacioglu and Akfirat 2015) but ask participants to report on a single, specific memory (e.g., a memory from one’s childhood). Table A1 in Appendix A presents the phenomenological properties tapped into by all the cited measures.

These two approaches have pros and cons, depending on the aim of the assessment. Scores from multi-item measures are inherently more reliable than single-item ones if a single memory is considered, but they are impractical to use with multiple memories due to their length. As a result, the information they provide is limited to the specific memory elicited and cannot be considered representative of the general and typical way in which the participant “remembers”, i.e., as a stable disposition. In single-item measures, the score on the dimension can be computed as a composite measure of the same item rated on multiple memories, each of which is elicited by a cue word that could be presented together with other cue words in a single session (e.g., Vannucci et al. 2020) or one per day (e.g., Vannucci et al. 2021). Such composite scores are reliable as much as scores on multi-item measures, but, given the different eliciting stimuli for the memories, they can be considered more informative about the extent to which each dimension is relevant in the participant’s autobiographical remembering in general. Hence, they can be interpreted as a stable disposition or individual difference regardless of the specific memory being evaluated. For example, a participant could obtain a high score on the AMCQ vividness and emotional valence scales when recalling a memory related to a romantic relationship experience. This does not imply that the autobiographical remembering of that participant is typically vivid and emotionally loaded, while this conclusion can be drawn based on a participant who rates a dozen or so memories as very vivid and emotionally loaded elicited by different cues. Recently, Rubin (2021) has supported this view of the properties of ABMs as reliable and stable individual differences with a somehow “hybrid” approach. In his study, participants attended two sessions in which they were presented with seven event cues (e.g., “during travel or vacation”, “from school or work”) and had to write a brief description of an event they remembered from school or work for each cue. After completing the description, the participants had to rate the phenomenological characteristics of their memory on 12 pairs of 7-point rating scales that corresponded to a dimension, thus allowing a multi-item assessment of the dimensions across multiple cues.

Other measures to assess the phenomenology of ABMs do not involve elicitation and subsequent ratings of dimensions but generally ask participants to indicate their agreement with statements about how they remember personal experiences. The Autobiographical Recollection Test (ART, Berntsen et al. 2019) is a general measure of the subjective experience individuals have of their memories that operationalizes seven of the dimensions assessed by the instruments mentioned above. Interestingly, while the correlations between the different phenomenological dimensions reported by Rubin (2021) ranged from null to .91, thus implying that at least some dimensions provide independent information, those reported by Berntsen et al. (2019) and by Matsumoto et al. (2022b) in the Japanese adaptation study of the ART were uniformly very strong (*r* ≥ .60) to the point that a single-factor model seemed a more parsimonious measurement model for the ART items. This single score provides a general measure of how much respondents think they remember their past well and focuses on the recollective experience of ABMs without distinguishing between dimensions.^1^

These conflicting results about the relationships among the different phenomenological dimensions are consistent with an issue recently noted by Talarico (2023), who observed that, despite the agreement on the different metacognitive constructs and recollective experience-related qualia assessed by the aforementioned instruments, the pattern of association between them is still not clear. Talarico (2023) provides an example of the association between vividness and the quantity and clarity of sensory/perceptual details. Although most scales consider these dimensions distinct, it is questionable whether participants would recognize this distinction as natural or evident.

In summary, a wide range of the phenomenological dimensions of ABMs can be assessed using several comprehensive measures. However, further research is needed on the inter-individual and intra-individual patterns of association of these dimensions, as suggested by Rubin’s (2021) findings. Recent studies in psychopathology advised against drawing inferences from cross-sectional covariance structures to individuals within the group, since associations between variables at the group level do not necessarily translate at the individual level (see, e.g., Bos et al. 2017; McNally 2021; Borsboom et al. 2021) unless the process is ergodic; that is, the mean and variance are the same for the group as for each individual member of the group (the “homogeneity condition”) and neither the mean nor the variance changes over time (the “stationarity condition”) (Molenaar and Campbell 2009). We suggest that future studies should investigate whether individual patterns of association between the phenomenological dimensions of ABMs differ from group patterns and, if so, whether they do so in degree and/or in kind, and whether these patterns change with time.

## 4. New Developments: The Phenomenology of the Retrieval Process 

In daily life, we often use expressions such as *“this memory just came to my mind; it arrived rapidly”*; *“he looked familiar to me; I was pretty sure I knew him, and I tried hard to remember where I had met him, but I couldn’t find this information in my memory”; “this memory surprised me; I didn’t know where it came from.”* In these sentences, we do not refer to the phenomenological properties of the retrieved memories (for example, vividness, clarity, richness of details, personal importance), namely, the final result of retrieval processes; but we just describe and share with other people our subjective experience of the retrieval process, “how” retrieving a memory was like. As pointed out by Moulin et al. (2023), autobiographical retrieval is “*inherently metacognitive*” (p. 11), because it is accompanied and shaped by the “*epistemic feelings*” (p. 5) experienced by those who remember. These feelings (e.g., how fluently a memory was retrieved) are metacognitive because they reflect “*fast-acting*” (p. 5) evaluations that are used by the person who remembers and that might guide the retrieval process. 

All the questionnaires described above were originally developed to provide a comprehensive assessment of the phenomenology of autobiographical remembering. They are mostly, if not entirely, made up of items related to the *quality of the remembered material* (e.g., vividness, richness of details, and emotional intensity) rather than the retrieval process. With the only exception of the question about ease of retrieval/feeling of effort, which is included in only a few of the measures (e.g., “*This memory just sprang to my mind when I read the instructions*” in MEQ and APAM, or “*I really had to search my ‘memory bank’ for this experience*” in MEQ), there are no questions in these instruments about the *subjective experience* of the *retrieval process*, that is, about the epistemic feelings experienced during the retrieval.

So, why has research on the phenomenology of autobiographical remembering mostly focused on the phenomenological characteristics of the final (memory) products and somehow neglected the phenomenology of the retrieval process? And which dimensions, if any, of the phenomenology of the retrieval have been started to be empirically addressed so far?

One of the reasons for this delay in research may be found in the theoretical models of ABM. Specifically, for a long time, the multifaceted nature of autobiographical retrieval has been under-recognized by cognitive psychologists. According to the self-memory system model of ABM (Conway 2005; Conway and Pleydell-Pearce 2000; Haque and Conway 2001), autobiographical remembering would occur primarily through a generative (i.e., active top-down search process), effortful, and time-consuming process (usually taking up to 10–12 s), whereas a direct (i.e., a memory directly popping into mind in response to a cue), effortless, and fast retrieval process would be relatively uncommon. Following this model, generative retrieval has been considered the default process for personal memories generated in standard word-cueing tasks, and the contribution of other routes through the autobiographical system has been largely overlooked (Markostamou et al. 2023).

Only over the past decade have studies provided evidence that direct retrieval often occurs in the context of voluntary autobiographical remembering; specifically, studies have shown that direct retrieval is as frequent as the generative one in standard word-cueing paradigms (e.g., Barzykowski and Staugaard 2016; Harris et al. 2015; Harris and Berntsen 2019; Mace et al. 2017; Uzer 2016), or it is even more frequent when concrete word-cues or personally relevant cues are used (Uzer 2016; Uzer and Brown 2017; Uzer et al. 2012; but see also Mace et al. 2021, for a discussion on the prevalence of direct retrieval). 

These findings have stimulated research on the phenomenology associated with the two qualitatively distinct retrieval mechanisms. In this regard, studies have found that memories subjectively evaluated as *directly* retrieved (i.e., suddenly and effortlessly retrieved) are reported to be clearer and more vivid (Barzykowski and Staugaard 2016; Harris and Berntsen 2019; Harris et al. 2015), more rehearsed (Harris and Berntsen 2019; Harris et al. 2015), of greater personal importance (Barzykowski and Staugaard 2016; Harris and Berntsen 2019; Harris et al. 2015), and they are more likely to have a field perspective (Harris et al. 2015) compared to *generatively* retrieved (i.e., retrieved after effortful search) memories. Globally, these results on the phenomenology of direct and generative retrieval demonstrate that there is a consistent association between one phenomenological dimension of the retrieval process itself (i.e., the subjective feeling of effort) and the phenomenological properties of the final outputs (e.g., vividness, clarity, and personal importance of memories), raising the question about the nature of this relationship. On the one hand, the phenomenological properties of memories and events might affect the experience of retrieval, so personal memories and events that are highly accessible (i.e., more vivid, clearer, and emotionally intense) are expected to be more frequently recalled in a direct/effortless rather than generative/effortful fashion since they do not require additional elaboration and reconstructive effort (Conway 2005). On the other hand, however, the retrieval process may affect the final output, so that effortless, bottom-up associative retrieval processes might lead to a richer experience of remembering, finally resulting in highly accessible retrieved memories. 

The scientific interest in the retrieval process has also been stimulated by the advances in the experimental investigation of involuntary ABMs^2^ (IAMs) that have been overlooked in experimental cognitive psychology for several decades (see Berntsen 2010; Berntsen 2021; Kvavilashvili et al. 2020). The successful development and employment of experimental paradigms to induce and examine involuntary memories in a laboratory setting (e.g., Barzykowski and Niedźwieńska 2016; Berntsen et al. 2013; Cole et al. 2016; Hall et al. 2014; Schlagman and Kvavilashvili 2008; Mazzoni et al. 2014; Vannucci et al. 2014) have allowed the comparison of voluntary and involuntary retrieval under the same well-controlled conditions. 

In terms of phenomenology, direct comparisons between the two types of retrieval have found that IAMs are more phenomenologically sound compared to their voluntary counterpart: Specifically, involuntary memories were found to be more specific (e.g., Cole et al. 2016; Schlagman and Kvavilashvili 2008; Vannucci et al. 2016), detailed, vivid, more frequently rehearsed (e.g., Barzykowski and Staugaard 2018; Vannucci et al. 2016), more personally relevant and more recent (e.g., Barzykowski and Staugaard 2018), and associated with a more intense emotional reaction at retrieval/being more impactful on current mood compared with voluntary memories (e.g., Cole et al. 2016; Vannucci et al. 2016). In addition, involuntary memories were reported as more pleasant, rehearsed, important, and relevant to the current life situation compared to both generative and direct voluntary memories (Barzykowski and Staugaard 2016). According to the retrieval threshold account (Barzykowski and Staugaard 2016, 2018), when someone is not intentionally engaged in memory retrieval (i.e., not being in a retrieval mode), a memory would need to be phenomenologically strong (e.g., rehearsed, emotional, important) to draw one’s memory-related attention and therefore pass the awareness threshold and enter consciousness.

Collectively, these studies show that both the retrieval effort and the intention to remember can affect the phenomenology of autobiographical remembering. However, some limitations should be taken into account when considering these results. First, in different studies, different subsets of phenomenological dimensions have been examined, and no studies so far have provided a comprehensive assessment of the phenomenology of IAMs by using comprehensive measures as the ones described in Section 3 originally developed to examine voluntary ABMs. Furthermore, the different retrieval processes can be accompanied and shaped by different epistemic feelings, namely, different evaluations of the retrieval itself. As reported above, subjective evaluation of effort/ease of recall has been found to be quite relevant in voluntary retrieval, distinguishing between direct and generative memories, and recent evidence confirmed that this subjective experience is also reflected in objective measures associated with the two kinds of retrieval (e.g., pupil size in Janssen et al. 2021). 

To this regard, very recently, researchers in cognitive psychology (e.g., Moulin et al. 2023) and philosophers of memory (e.g., Perrin et al. 2020; Perrin and Sant’Anna 2022) have directly called for a more systematic empirical investigation of the phenomenology of the retrieval process itself, as has been completed with the phenomenological characteristics of ABMs. 

Such a novel theoretical approach stimulates and requires an empirical examination and the development of methodological tools that might be different from those used so far. A first step could be a broad exploration of the phenomenological space of retrieval, that is, identifying the different experiences or epistemic feelings that participants may have during the retrieval of ABMs. Although self-report questionnaires could be used, an investigation using in-depth phenomenological and qualitative methodology could provide more insights.

For instance, Oblak et al. (2022) have recently used a constructivist grounded theory approach (Charmaz 2014) to investigate the subjective experience of their participants during a visuospatial working memory task. This method aims to provide a detailed outline of the structure of a certain phenomenon. As a result, it seeks to characterize as many distinct experiences related to a phenomenon as is feasible. In order to enable comprehensive explanations, constructivist grounded theory collects as wide-ranging information as it can from as many sources as it can. Thus, using a variety of visuospatial working memory tests, Oblak et al. (2022) were able to collect detailed qualitative data from a diverse sample of participants with varying ages and educational backgrounds. Their results revealed two major categories of experiential dimensions: phenomena at the forefront of consciousness and background feelings. The first refers to elements of experience that hold a prominent position in the consciousness of participants and may be easily accessed for reflection, even without formal instruction on how to observe and articulate one’s experiences. These phenomena encompass a range of techniques employed in the resolution of visuospatial working memory tasks, along with metacognitive experiences and instances of mind wandering. The second dimension concerns the overall impression of the experience (e.g., physical sensations, emotional climate, mood) and might be harder to access in conscious reflection. 

Moulin et al. (2023) have identified some dimensions of the retrieval experience, such as the feeling of familiarity, the feeling of agency, and the feeling of fluency. The feeling of familiarity is a state in which the individual can only report whether something (an image, an object, a person, etc.) has been encountered before, i.e., it reminds them of “something”, while the feeling of agency is the sensation of being the initiator of the retrieval process. The feeling of fluency is the subjective experience of how readily a memory comes to mind, regardless of other subjective variables and theoretical entities (e.g., vividness, accuracy, and meaning for the self) that can be assessed once memories have been retrieved. When the retrieval process is fluent, the sense of agency is reduced, and there is often a sense of familiarity that is not an inherent feature of something that has been seen before. Investigating such feelings holds the potential to contribute to understanding the phenomenology of the retrieval process, but, similar to the background feelings of Oblak et al. (2022), it appears methodologically challenging. 

**Figure 1:**
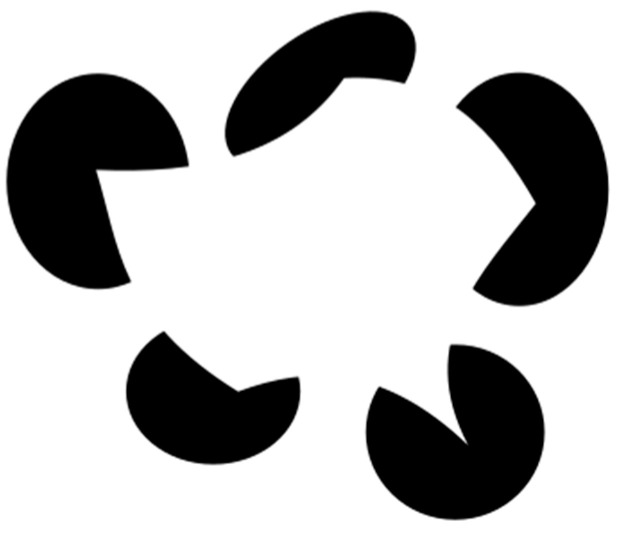
Example of illusory contours.

Interestingly, the dimensions suggested by Moulin et al. (2023) resemble a class of phenomena studied by experimental phenomenology, an approach that has a long history in the study of visual perception. For example, the subjective experience of memory fluency is not Tulving’s *autonoetic consciousness* or Perrin et al.’s *feeling of pastness*, since these terms refer to the experience once the memory has occurred. When we evaluate the vividness or coherence of memory, we are *thinking* about it, while when we evaluate its fluency, we are sort of *seeing* it, in parallel with Kanizsa (1979)’s distinction in visual perception. As a result, our memory can be tricked by “illusions” just as well as our visual system, as in the case of *déjà vu*. For example, when entering a place, one may feel strongly that they have been there before, even though they are aware that this is their first visit. This process resembles, for instance, that of illusory (or subjective) contours in perception, which are optical illusions that give the impression of an edge without really changing the luminance or color of that edge (Figure 1).

Explaining why this perceptual effect occurs is beyond the scope of this work, but one classic explanation is Kanizsa’s “causal hypothesis” (see, e.g., Kanizsa 1976, 1979), which assumes, in line with Gestalt theory’s dynamic model of object formation, that this process starts with phenomenal incompleteness, or “open figures”, which is a basic requirement for form improvement. Despite the fact that both locations offer the same level of visual stimulation, the area within the subjective contours appears to be an opaque surface superposed on the other figures and brighter than the background (Figure 1).

Urquhart et al. (2021) proposed that déjà vu could be the outcome of a clash in mental evaluations and a conflict in appraisals in memory, while Cleary et al. (2012) suggested that it could be caused by environmental cues that have some undetected conceptual or perceptual overlap with stored representations. This gives a sense of fluency by making the environment and/or situation feel familiar, as if one had *actually* been there before. As a result, the subjective experience of how readily a memory comes to mind is something that exists at the moment of observation and thus appears consistent with the definition of a perceptual event in experimental phenomenology (see, e.g., Bianchi and Davies 2019). According to experimental phenomenology, the “mother of all things” (Bianchi and Davies 2019, p. 340) is not what the eye has seen but what, objectively, has been in front of the observer in the act of observation, that is, the observer’s immediate and direct connection to objects, events, properties, and relations. Although the reporting of the phenomenological characteristics of the memories cannot be considered an act of observation, the very process that leads to the happening of the memory does; indeed, in the Gestalt tradition, the act of suddenly remembering something is considered one of the diverse psychological experiences that originate simultaneity and constitute autonomous and closely integrated blocks, complex indivisible “Gestalten” (see, e.g., Lewin 1926).

From the point of view of “classical” phenomenology, the act of remembering can be considered as an act of reflection, which, in turn, is an act of thought that conceives thoughts as acts and becomes aware of them (Levinas 1969). The very act of recalling brings to light the evidence of the gaze of consciousness, as it implies turning attention to the flowing thought and paying attention to it (Husserl [1913] 1982). In other words, the I “directs itself” toward its own lived experiences. Although memories are not present to the gaze as perceptual events, the process of recalling them is something that can be “seen” when reflection is focused on it, making it an object for the individual (Husserl [1913] 1982).

Just as the perception of illusory contours can be induced using displays such as the one in Figure 1, it has been shown that déjà vu can be induced, too, using, for instance, the recognition without identification paradigm (Cleary 2008). In summary, this paradigm comprises two phases. In the first, participants are presented with line drawings of unique scenes and asked to study and learn them. In the second, they are presented with other scenes that nonetheless share a large perceptual similarity with the previous ones and thus generate a feeling of familiarity. When this feeling is combined with the failure to produce a name (i.e., recognition without identification), the probability of the occurrence of déjà vu significantly increases.

Recently, Barzykowski and Moulin (2023) argued that déjà vu and IAMs could be thought of as two products of the same mechanisms of involuntary retrieval and thus lie on a continuum. Although déjà vu lacks access to memory content and the feeling of familiarity is judged as implausible, IAMs are accompanied by recognized memory content. Barzykowski and Moulin (2023) call for the investigation of “what it is like to have IAMs and déjà vu” (p. 15), as it is still not clear whether only the presence (or lack thereof) of the content makes them distinct or whether there are more differences in the way they are phenomenologically experienced and described by the participants, beyond intentionality, plausibility, and feeling of fluency. 

Although there are methods to induce them experimentally, their neurological bases are known and questionnaires and diaries can be used to collect data on their products, the very process has yet to be understood from a phenomenological point of view. The experimental phenomenology approach holds that while a noetic (i.e., cognitive) structure can act upon observable facts once they are remembered, it cannot upon what is being observed. In Bozzi’s definition, “phenomenal reality” is the world that individuals inter-observe and inter-subjectively share, different from the cognitive integrations and interpretations that they apply to it (Bianchi and Davies 2019). In visual perception, this allows the application of the interobservation method, i.e., a method of observation that is based on how people negotiate perception in a social setting, where participants can talk to each other and to the experimenters to get a better sense of their phenomenological experience. Participants are invited to try and understand each other, reformulate their impressions, and provide any kind of verbal and non-verbal description in order to reach a common and richer view of the dynamics of phenomena (Bozzi 1978; Bianchi and Davies 2019, chap. 10). 

It should be noted that this method is not a mere “introspection”, that is, a way of capturing what a given individual is currently experiencing or thinking about, since the phenomenological field of research does not concern private thoughts but intersubjectively accessible modes of appearance (see, e.g., Zahavi 2003). Interobservation is not only interested in subjective experiences and their structures but also in how much they are representative of common experiences in order to grasp the invariant self-organizing structure of the experience (Gallagher and Sørensen 2006).

Instead of being challenged to fit into predefined descriptive categories, participants are encouraged to create their own descriptions. A methodological step known as “phenomenological reduction” is carried out by the participants, who are required to focus only on their own experiences and their conscious appearance. All beliefs, opinions, and theories about what that experience is are to be excluded, including naive metaphysical and/or introspectivist views about the nature of the mind or even the existence of a mind at all. This strategy aims to prevent the particularistic objectives of the experimenter or the participant from skewing descriptions. Moreover, establishing the veracity of a claim is not the participant’s responsibility. The only instructions given to the participants are to focus on and explain their personal experiences without generating theories or opinions about them. This approach to collecting first-person data does not necessarily result in third-person quantitative data. Instead, qualitative similarities between the reports of one subject and those of other subjects can be compared in an effort to identify the invariant patterns of experience under particular experimental conditions (Gallagher and Sørensen 2006).

Although déjà vu and IAMs are basically private facts whose observation cannot be shared, it would be interesting to test, for example, whether the same déjà vu in multiple participants can be (experimentally) induced, thus allowing the observation of a “common fact” and the application of interobservation. In other words, there would be a need to combine an existing experimental approach (e.g., the recognition without identification paradigm by Cleary et al. 2009) with a focus on the shared, lived experience. The added value of this method would be the availability of descriptions of participants’ experiences in their own words, capturing the richness of their phenomenological accounts. The data obtained with this approach could help shed light on the very process underlying déjà vu and IAMs, the essential elements, and the subjective meaning attributed to the phenomenon, over and above those already found in previous studies with different research methods, hopefully providing a comprehensive answer to Barzykowski and Moulin (2023)’s question.

## 5. Conclusions

As we reviewed above, important conceptual and methodological advancements have been made in the research field on the phenomenology of autobiographical remembering over the past decades. First, research on the phenomenological properties of ABMs has clearly shown that phenomenology is quite relevant to distinguishing between different kinds of memory, and it is also modulated by individual differences in personality and cognition in those who remember. Second, and relatedly, the well-documented importance of the phenomenology of ABM has prompted the development of psychometrically sound instruments that can accurately and comprehensively measure the multifaceted dimensions of the phenomenology according to the aim of the assessment.

In recent years, increasing attention has been paid to the investigation of the phenomenology associated with different types of retrieval (i.e., generative, direct, and involuntary), and this development has stimulated a more general interest in the issue of the phenomenology of the autobiographical retrieval process itself, that is, the epistemic feelings that participants may have during the retrieval of ABMs.

Indeed, studying phenomenology from the point of view of the retrieval process could provide a significant contribution to the understanding of the mechanisms and states involved during this process. As recently suggested (Moulin et al. 2023), epistemic feelings experienced by those who remember, while remembering, not only accompany but are also used and guide the cognitive process of retrieval. So far, a systematic investigation of the different dimensions of the phenomenology of the retrieval process is still missing; indeed, this kind of investigation also raises questions about the most suitable methodological tools.

Here, we suggest that comparing these subjective retrieval process experiences to those under investigation by experimental phenomenology opens up new perspectives on what it is like to experience them. Experimental phenomenology aims at discovering the structural laws of experience and focuses on the empirical knowledge of the real world of our living experience. The emotional and expressive qualities of autobiographical phenomena could be conceived as structural laws of the remembering process and should be considered in all their complexity and richness, even those, such as déjà vu, that can naively appear as “bugs” of the system.

## Data Availability

Not applicable.

## Notes

1 For the sake of completeness of information, other measures have been developed taking a different approach from the ones mentioned here, such as the Awareness of Narrative Identity Questionnaire (ANIQ, Hallford and Mellor 2017) and the Survey of Autobiographical Memory (SAM, Palombo et al. 2013). The ANIQ is a measure of the awareness of narrative identity and the perception of the global coherence of ABMs in terms of temporal order, causal associations, and themes, and it focuses on how respondents generally use their personal memories rather than trying to relate them to specific circumstances or experiences. The SAM has a wider scope and taps into episodic, semantic, spatial, and prospective (future-directed) aspects of memory and mental images as four different and relatively independent dimensions of self-reported mnemonic characteristics. Despite the name, the content of the items is only marginally related to ABMs, except for the episodic scale: The semantic scale deals with memory for past information, the spatial scale with the ability to remember places and routes, and the future scale with the ability to imagine and visualize future events.

2 In some empirical studies and theoretical papers, direct and involuntary retrieval have been conflated, and they have been treated as identical processes (e.g., Brewin et al. 2010; Conway and Pleydell-Pearce 2000). However, as discussed in several recent papers (e.g., Barzykowski and Staugaard 2018; Harris and Berntsen 2019; Harris et al. 2015; Mace et al. 2021; Matsumoto et al. 2022a), the two types of retrieval show similarities in terms of the mental operations involved (i.e., both direct and involuntary retrieval are not strategic and the memory arises quickly and effortlessly), but also differences in terms of their defining characteristics. The defining feature of involuntary retrieval is its lack of intentionality (i.e., retrieving without any conscious or deliberate attempt to retrieve, without any explicit memory prompt), whereas the defining feature of direct retrieval is its lack of effort. Moreover, at a methodological level, the paradigms that have been employed to examine and compare direct and generative retrieval require voluntary retrieval; that is, participants are instructed to retrieve a memory in response to a cue, they follow an explicit memory prompt (e.g., a standard word-cue paradigm), and they enter the mental state of remembering (i.e., the retrieval mode). On the contrary, in the paradigms used to examine involuntary retrieval, participants are not informed or asked to recall any memories, and memories come to mind without any deliberate intention to retrieve them. Moreover, IAMs often occur while people are doing other undemanding and monotonous activities, and attention is not focused on retrieval, which is not the task at hand. For this reason, in some situations (i.e., the higher attentional load associated with the ongoing task), IAMs may also go unnoticed (e.g., Vannucci et al. 2019; Vannucci and Hanczakowski 2023). As reported in the study by Barzykowski and Staugaard (2018), direct and involuntary memories also differ in terms of their phenomenological properties, suggesting that the lack or presence of intention during retrieval affects the retrieval itself and its final outputs.

## Appendix A

**Table A1:** Measures for the assessment of phenomenological characteristics of autobiographical memories.

Measure	Number of Items	Elicitation Method	Answer Scale	Dimensions
Memory Characteristics Questionnaire (MCQ, Johnson et al. 1988)	39	Thinking of an actual event (e.g., social occasion) and an imagined event (e.g., dream)	7-point rating scale	Clarity, color, visual detail, sound, smell, touch, taste, vividness, event detail, order, complexity, realism, location, setting, objects (spatial), people (spatial), time, year, season, day, hour, event duration, tone (negative/positive), participant, seeming implications, actual implications, remembered feeling, felt (negative/positive), felt intense, current intensity, remembered thoughts, self-revealing, overall memory, events before, events after, doubt/certainty, covert rehearsal, overt rehearsal
Autobiographical Memory Questionnaire (AMQ, Rubin et al. 2003)	15	30 cue words (e.g., “candy”)	7-point rating scale	Reliving, back in time, remember/know, real/imagine, persuade, accurate, testify, see, setting, spatial, hear, talk, in words, story, emotions, importance, rehearsal, once/many, merged/extended, age of memory
Memory Experiences Questionnaire (MEQ, Sutin and Robins 2007)	57	Writing about a general self-defining memory and the earliest childhood memory	5-point agreement scale	Vividness, coherence, accessibility, time perspective, sensory detail, emotional intensity, visual perspective, sharing, distancing, valence
Autobiographical Memory Characteristics Questionnaire (AMCQ, Boyacioglu and Akfirat 2015)	63	Recalling a memory from childhood and a memory related to one’s romantic relationship experiences	7-point agreement scale	Vividness, belief in accuracy, place details, sensory details, accessibility, sharing, observer perspective, field perspective, narrative coherence, recollection, emotional valence, emotional intensity, emotional distancing, preoccupation with emotions, time details, emotional persistence, visceral reactions, personal implication of the event
Autobiographical Recollection Test (ART, Berntsen et al. 2019)	21	General questions about the way participants remember events from their past	7-point agreement scale	Vividness, coherence, reliving, rehearsal, scene, visual, life story
Phenomenology of Autobiographical Memory questionnaire (APAM, Vannucci et al. 2020; web-based version in Vannucci et al. 2021)	25	12 cue words (e.g., “love”) presented in a single session (original version) or 7 cue words presented one per day (web-based version)	7-point rating scale	Clarity, color, vividness, visual detail, sound, smell, touch, taste, reliving, hearing in mind, seeing in mind, setting recall, remembering rather than just knowing, rehearsal, coherence, confidence in accuracy, ease of recall, participant/observer, felt, remembered feeling, feeling, having changed as a person since, talking, turning point, doubt/certainty
Rubins Rating Scales (Rubin 2021)	24	Event cues (e.g., “a mistake”)	7-point rating scale	Reliving, vividness, belief, visual, scene, contents, specific time, auditory, coherence, centrality, rehearsal, emotion
